# Non-invasive evaluation of renal structure and function of healthy individuals with multiparametric MRI: Effects of sex and age

**DOI:** 10.1038/s41598-019-46996-x

**Published:** 2019-07-23

**Authors:** Xue-Ming Li, Ling Yang, Jing Reng, Guo-Hui Xu, Peng Zhou

**Affiliations:** 1 Department of Radiology, Sichuan Cancer Hospital & Institute, Sichuan Cancer Center, School of Medicine, University of Electronic Science and Technology of China, No. 55, Lane 4, RenMin Road (South), Chengdu, Sichuan 610041 China; 2 Department of Radiology, West China Hospital, Sichuan University, 37# Guoxue Street, Chengdu, Sichuan 610041 China

**Keywords:** Diagnostic markers, Kidney

## Abstract

Clinically, when applying multiparametric magnetic resonance imaging (MRI) examinations in renal diseases, assessment of renal structure and function has to account for age- and sex-related effects. The aim of this study was to investigate the influence of age and sex on multiparametric MRI assessment of renal structure and function in healthy human beings. Studies on 33 healthy volunteers were performed using multiparametric MRI on a 3.0-Tesla MR scanner, including T1-weighted imaging, blood oxygen level-dependent MRI (BOLD MRI), diffusion-weighted imaging (DWI) and diffusion tensor imaging (DTI). Our results revealed that the mean renal cortical thickness (RCT), ratio of cortex to parenchyma (CPR), and cortical R2* values were higher in males than in females. The cortical R2* value was higher in older group than in younger group (18.57 ± 0.99 vs 17.53 ± 0.58, p = 0.001); there was no significant difference in medullary R2* between the older and younger groups (38.18 ± 2.96 vs 36.45 ± 2.47, p = 0.077). The parenchymal thickness (PT) and medullary fractional anisotropy (FA) were lower in older group than in younger group (1.547 ± 0.06 vs 1.604 ± 0.05, p = 0.005 and 0.343 ± 0.03 vs 0.371 ± 0.03, p = 0.016, respectively). Pearson’s correlation analysis showed that PT and medullary FA were inversely related with age (r = −0.483, p = 0.004; r = −0.446, p = 0.009) while cortical R2* values was positively related (r = 0.511, p = 0.002, respectively). The medullary apparent diffusion coefficient (ADC) value had a significant association with PT (r = 0.359, p = 0.04). This study indicated that multiparametric renal MRI parameters are age and sex dependent.

## Introduction

The kidney is an intricate organ, which plays an important role in ensuring acid-base balance, regulating electrolytes and blood pressure as well as filtering blood to remove waste substances (e.g. urate, urea, toxins) from the body. These physiological activities utilize oxygen and heavily depend on water molecule transporting ability of the kidney, which is strikingly different in renal cortex and medulla due to different vascular and tubular arrangement^1^. The renal medulla consumes more oxygen in the thick ascending limb by the Na-K ATPase activity than the cortex, which suggests hypoxia of the medulla in normal conditions^2^.

A normal aging kidney is accompanied by structural deterioration and progressive decline in renal function. Morphologically, the aging kidney shows a decrease in cortical mass and total nephron size and number, increase in arteriosclerosis, glomerulosclerosis, tubular atrophy, interstitial fibrosis, tubular diverticula, and to a lesser extent nephron hypertrophy^3,4^. The cortical atrophy roughly reflects decreased number of functioning nephrons, and few glomeruli appear sclerosed under the age of 40^4^. In terms of renal functions, there is increased renal vascular resistance and filtration fraction, reduced renal blood flow (RBF), glomerular filtration rate (GFR), urine concentrating capacity and hormone secretion with age^3,4^. Although the GFR decline usually begins from mid 40 s, the decline rate varies with gender, race, and burden of comorbid disease^4^.

Morphological changes of diffuse renal diseases can be detected only at advanced stages, which often leads to a delay in adequate treatment. Laboratory parameters, such as serum creatinine, cystatin C and urinary albuminuria leakage, are commonly used for providing renal filtration information. However, these parameters are insensitive to significant subtle changes. In the meantime, potential toxicities associated with contrast media used to measure GFR may restrict repetitive examinations within a short period^5^. As 3.0 T MR systems became more and more commonly used in the clinical field, many noninvasive MR sequences are being used in daily scanning to acquire information with higher signal-to-noise ratio and are suitable for kidney examination with no Gd-based contrast agent exposure^6^. Many studies using single MRI techniques have been used to detect alterations in intrarenal microstructure, oxygenation or perfusion in patients^7–10^. However, it is difficult to determine which are the optimal MRI measures or combinations of measures for a given biological process. Multiparametric MRI including conventional T1-weighted imaging, blood oxygen level-dependent (BOLD), diffusion weighted imaging (DWI) and diffusion tensor imaging (DTI), which combines multiple MRI biomarkers, is likely to be more effective at assessing biophysical tissue properties and providing insight into different pathological processes *in-vivo* in a single scan session. It is therefore necessary to establish the normal values of related parameters and their relevant influencing factors in healthy population before clinical application. However, it has only been performed in limited studies that analyze the impact of sex and age^11–15^. Thus, the objective of this study was to investigate the influence of age and sex on renal structure and function in healthy human beings with multiparametric MRI examination on a 3.0 T MR system.

## Results

Table 1 provides demographic details of the participants, which are divided into younger (<40 years) and older (>40 years) age groups and male and female groups for comparison.

**Table 1:** Characteristics of healthy participants by age and gender.

Risk factors	Sex Group (N = 33)		P-value	Age Group & (N = 33)		P-value	ALL Mean ± SD
	Females (n = 17)	Males (n = 16)		<40 (n = 19)	≥40 (n = 14)		
Age (years)	37.9 ± 16.0	39.0 ± 15.9	0.85	25.8 ± 2.0	55.6 ± 7.4	<0.001*	38.5 ± 15.7
Height (m)	1.59 ± 0.06	1.69 ± 0.07	<0.001*	1.64 ± 0.092	1.63 ± 1.065	0.059	1.64 ± 0.08
Weight (kg)	54.1 ± 9.5	62.8 ± 8.1	0.002*	57.9 ± 7.3	58.9 ± 10.1	0.752	58.30 ± 8.45
BMI (kg/m^2^)	21.48 ± 2.38	22.04 ± 2.6	0.519	21.46 ± 2.1	22.15 ± 2.92	0.435	21.75 ± 2.46
Systolic BP (mmHg) (mmHg)	123.6 ± 5.4	125.3 ± 7.0	0.401	122.1 ± 4.1	127.6 ± 6.3	0.004*	124.42 ± 5.79
Diastolic BP (mmHg)	71.0 ± 5.5	72.4 ± 7.0	0.516	68.5 ± 4.7	76.1 ± 5.4	<0.001*	71.70 ± 6.22

BMI: Body mass index; BP: Blood pressure. & Nine participants were male and 10 female in the group of age <40; and both male and female were 7 in the group of age ≥40.

**Figure 1:**
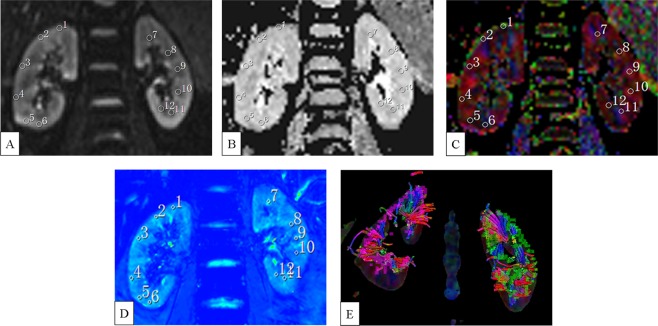
Coronal MR images of a 27-year old female. Six ROIs were drawn from upper to lower pole in the cortex and medulla respectively on DW (b = 0) image (**A**). The corresponding ROIs’ values in ADC map (**B**) and FA color map (**C**) were acquired automatically. Similarly, six ROIs were drawn from upper to lower pole in the cortex and medulla respectively on T2* map (**D**). The reconstructed FA Tractography of both kidneys (**E**) shows the orientation of medullary tubules. Note: Number 1 to 6 represent ROIs in the cortex, and number 7 to 12 in the medulla.

**Figure 2:**
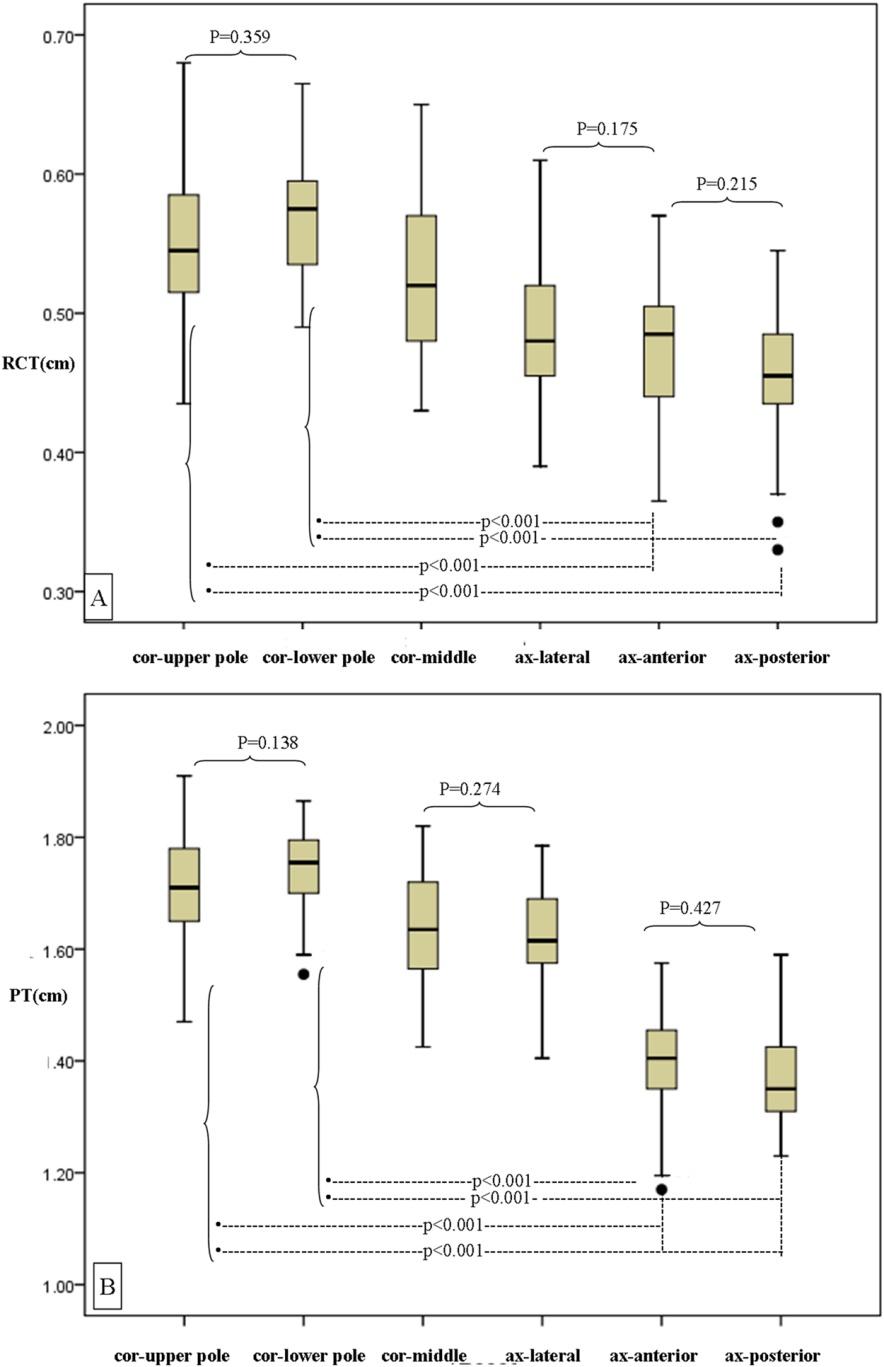
Comparisons between different areas of RCT and PT in the kidney using one-way ANOVA with Student-Newman-Keuls test. (**A**) RCT in different area of the kidney are significantly different from each other (p < or = 0.044). except comparisons between coronal upper and lower pole, axial lateral and anterior area, axial. anterior and posterior area. And RCT of all the coronal areas are significantly higher than that of axial areas, respectively (all p < 0.007). (**B**) PT in different area of the kidney are significantly different from each other (p < or = 0.001) except comparisons between coronal upper and lower pole, coronal middle and axial lateral area, axial anterior and posterior area. And PT of both coronal upper and lower poles are significantly higher than that of axial anterior and posterior areas, respectively (all p < 0.001). Note: RCT, renal cortical thickness; PT, parenchymal thickness.

Multiparametric MR images and ROI locations are demonstrated in Fig. 1. Six ROIs were drawn from upper to lower pole in the cortex and medulla respectively on DW (b = 0) image (Fig. 1A), and the corresponding ROIs’ values in ADC map (Fig. 1B) and FA color map (Fig. 1C) were acquired automatically. Similarly, ROIs were drawn from upper to lower pole in the cortex and medulla respectively on T2* map (Fig. 1D). The reconstructed fractional anisotropy (FA) tractography (Fig. 1E) of both kidneys shows the orientation of medullary tubules orienting to the renal hilum. There were no differences observed between the renal cortical thickness (RCT), parenchymal thickness (PT), ratio of cortex to parenchyma (CPR), R2*, apparent diffusion coefficient (ADC) and FA values of the left and right kidney. Figure 2 demonstrates the comparisons of RCT and PT in different areas of the kidney, which shows that RCT and PT of both coronal upper and lower poles are significantly higher than that of axial anterior and posterior areas, respectively (all p < 0.001).

Table 2 provides the MRI results for both gender and age groups. The results show that the RCT, CPR and cortical R2* values of men were significantly higher than that of women (p = 0.003, 0.007 and 0.024, respectively). The ratio of RCT and PT by body mass index (BMI) of the patient. revealed no significant difference (all P > 0.1) between gender. The cortical R2* value was significantly higher and PT and medullary FA values significantly lower in the older group compared to the younger group (p = 0.001, 0.005 and 0.016, respectively). There was no significant difference of either medullary R2* or cortical FA between the older and younger groups (p = 0.077 and 0.421, respectively). In addition, the mean medullary R2* and FA values were higher and medullary ADC value lower than that of cortex with significance (all p < 0.001).

**Table 2:** Between-subject variability for multiparametric MRI measures in healthy participants.

Parameter		Sex Group		P	Age Group		P	Mean/Total
		female	male		age <40	age ≥40		
RCT (cm)	Axial	0.458 ± 0.041	0.488 ± 0.040	0.044^§^	0.481 ± 0.48	0.461 ± 0.33	0.191	0.473 ± 0.0428
	coronal	0.525 ± 0.028	0.571 ± 0.037	<0.001^§^	0.558 ± 0.04	0.533 ± 0.03	0.085	0.547 ± 0.040^※^
PT (cm)	Axial	1.436 ± 0.077	1.485 ± 0.055	0.045^§^	1.482 ± 0.06	1.43 ± 0.07	0.035^§^	1.46 ± 0.071
	coronal	1.687 ± 0.066	1.713 ± 0.065	0.268	1.726 ± 0.06	1.664 ± 0.06	0.006^§^	1.699 ± 0.07^※^
CPR	Axial	0.321 ± 0.022	0.331 ± 0.023	0.229	0.327 ± 0.024	0.325 ± 0.021	0.869	0.326 ± 0.02
	coronal	0.312 ± 0.009	0.334 ± 0.018	<0.001^§^	0.323 ± 0.02	0.321 ± 0.015	0.73	0.323 ± 0.02
RCT (cm)	Average	0.492 ± 0.032	0.529 ± 0.034	0.003^§^	0.519 ± 0.04	0.498 ± 0.03	0.1	0.510 ± 0.04
PT (cm)		1.562 ± 0.062	1.599 ± 0.053	0.074	1.604 ± 0.05	1.547 ± 0.06	0.005^§^	1.580 ± 0.05
CPR		0.317 ± 0.013	0.332 ± 0.018	0.007^§^	0.325 ± 0.02	0.323 ± 0.01	0.765	0.324 ± 0.02
Average RCT/BMI		0.023 ± 0.003	0.024 ± 0.003	0.253	0.024 ± 0.003	0.023 ± 0.003	0.124	0.024 ± 0.003
Average PT/BMI		0.073 ± 0.007	0.073 ± 0.007	0.966	0.075 ± 0.007	0.071 ± 0.007	0.065	0.073 ± 0.007
R2* (s^−1^)	cortex	17.62 ± 0.84	18.34 ± 0.90	0.024^§^	17.53 ± 0.58	18.57 ± 0.99	0.001^§^	17.97 ± 0.93
	medulla	36.33 ± 2.81	38.09 ± 2.54	0.069	36.45 ± 2.47	38.18 ± 2.96	0.077	37.18 ± 2.78^#^
ADC (x 10^−3^ mm^2^/s)	cortex	2.212 ± 0.16	2.229 ± 0.096	0.712	2.204 ± 0.17	2.243 ± 0.067	0.406	2.220 ± 0.13
	medulla	2.012 ± 0.14	2.019 ± 0.11	0.873	2.047 ± 0.14	1.974 ± 0.11	0.113	2.016 ± 0.13^#^
FA	cortex	0.203 ± 0.02	0.212 ± 0.019	0.171	0.210 ± 0.02	0.204 ± 0.02	0.421	0.207 ± 0.02
	medulla	0.359 ± 0.03	0.360 ± 0.04	0.909	0.371 ± 0.03	0.343 ± 0.03	0.016^§^	0.359 ± 0.03^#^

RCT: Renal cortical thickness; PT: Parenchymal thickness; CPR: Ratio of cortex to parenchyma; BMI: Body mass index.

^§^p < 0.05.

^※^<0.001 compared to the axial by independent sample t-test.

^#^<0.001 compared to the cortex by independent sample t-test.

**Figure 3:**
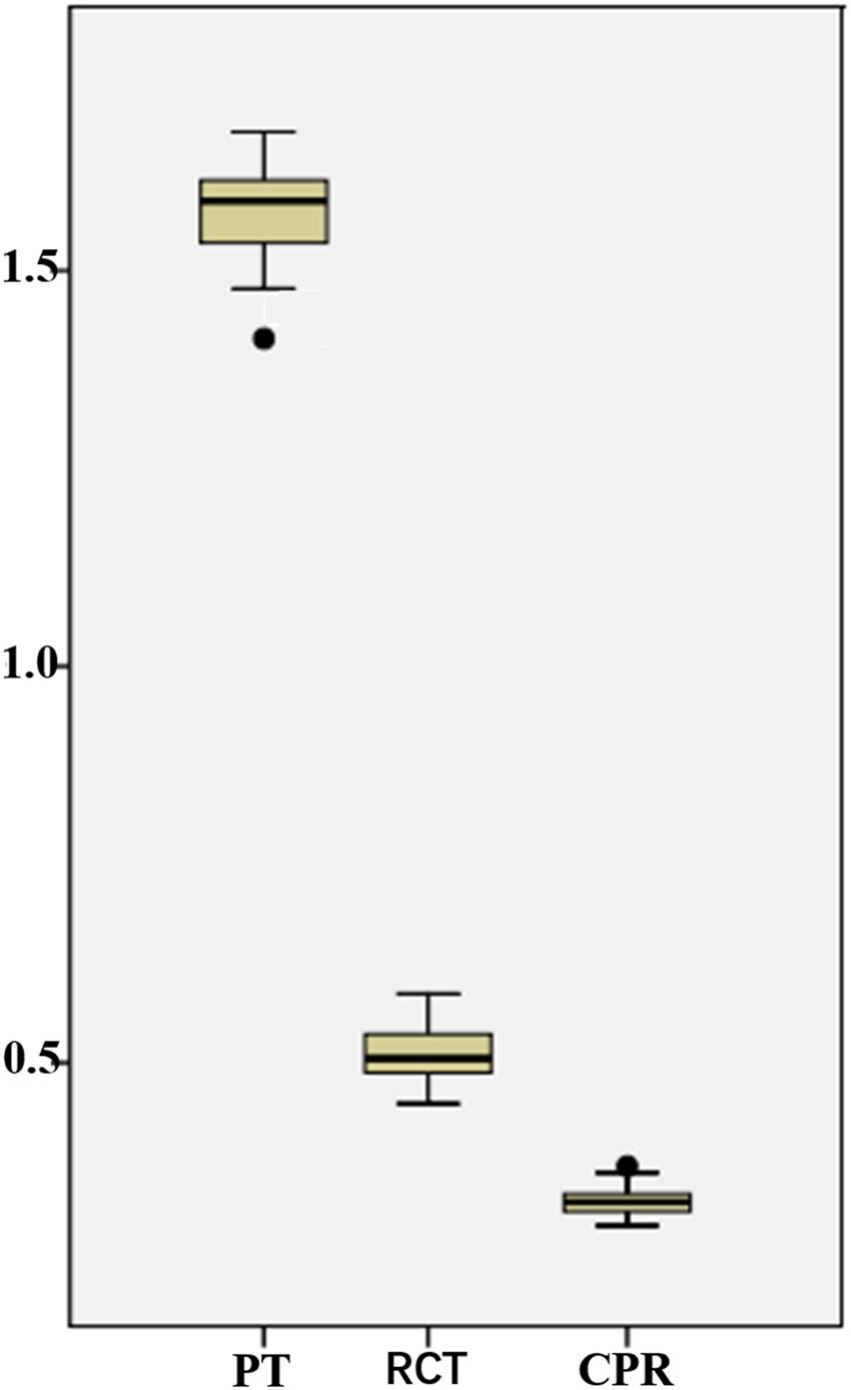
Box plots show PT, RCT and CPR in healthy subjects. Each box stretches from the 25th percentile at the lower edge to the 75th percentile at the upper edge; the median is shown as a line across the box. The dispersions of RCT and CPR are smaller than that of PT. The unit on y axis is centimeter for RCT and PT. Note: PT, parenchymal thickness; RCT, renal cortical thickness; CPR, ratio of cortex to parenchyma.

**Figure 4:**
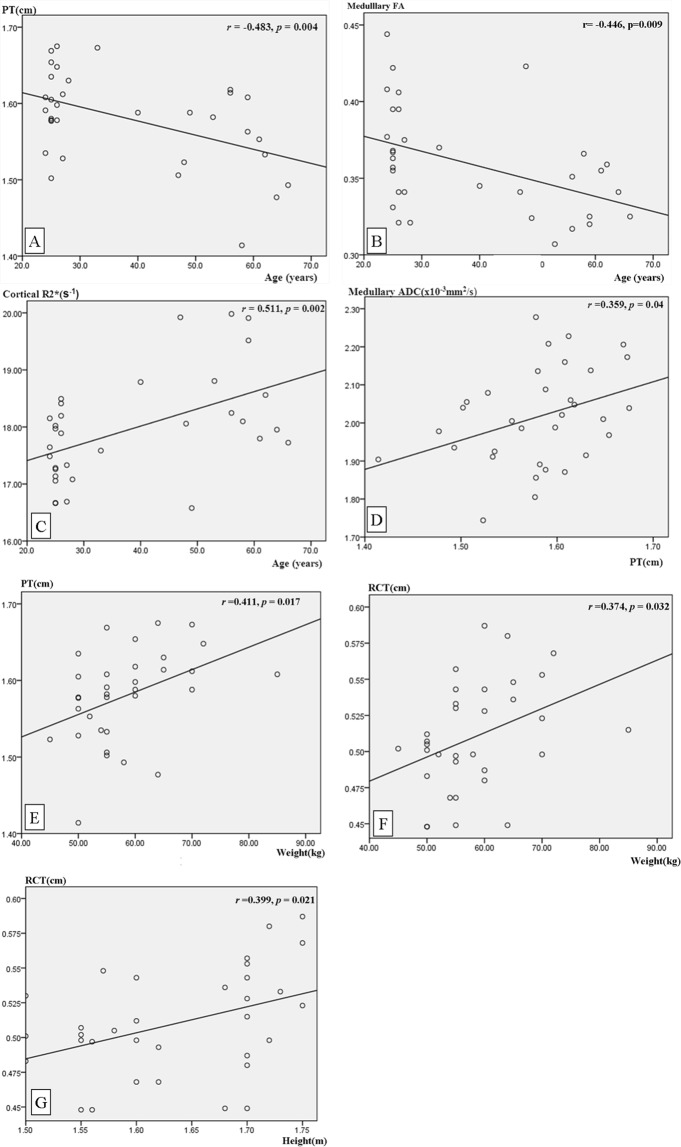
Correlation between MRI parameters and related factors. (**A**) PT versus patient age. A significant negative correlation was found between patient age and PT (r = −0.483 with p = 0.004). (**B**) Medullary FA versus patient age. A significant negative correlation was found between medullary FA and age (r = −0.446 with p = 0.009). (**C**) Cortical R2* versus patient age. A significant positive correlation was found between patient cortical R2* and age (r = 0.511 with p = 0.002). (**D**) PT versus medullary ADC. A mild positive correlation was found between PT and ADC (r = 0.359 with p = 0.04). (**E**,**F**) PT and RCT versus body weight. A significant and mild positive correlation was found between PT, RCT and body weight, respectively (r = 0.411, p = 0.017; r = 0.374, p = 0.032). (**G**) RCT versus height. A mild positive correlation was found between RCT and height (r = 0.399, p = 0.021). Note: PT, parenchymal thickness; FA, fractional anisotropy; ADC, apparent diffusion coefficient.

The mean values of RCT, PT and CPR are shown by box plot (Fig. 3), which demonstrates that the values of RCT and CPR showed smaller degree of dispersion than that of PT. Linear regression analysis (Fig. 4) demonstrates that there was a moderately negative correlation between PT and age (r = −0.483, p = 0.004), medullary FA and age (r = −0.446, p = 0.009); a moderately positive correlation between cortical R2* value and age (r = 0.511, p = 0.002), PT and weight (r = 0.411, p = 0.017); and a mild linear correlation between PT and medullary ADC value (r = 0.359, p = 0.04), RCT and weight (r = 0.374, p = 0.032) as well as RCT and height (r = 0.399, p = 0.021). In addition, the cortical and medullary R2*, ADC and FA were not correlated with height, weight or BMI; both PT and RCT were not correlated with BMI; and PT was not correlated with height.

The between session and inter-observer variability of the MRI measurements were also analyzed. The ICCs (intra-class correlation coefficients) and CoV (coefficient of Variation) for between session variability were 0.76 to 0.88 and 3.7% to 13% respectively, 0.683 to 0.790 and 4.4% to 10.5% for inter-observer variability (Table 3).

**Table 3:** Inter-observer and between session variability of multiparametric MRI measurements in healthy participants.

Parameter		Inter-observer variability		Between session variability	
		ICC	CoV (%)	ICC	CoV (%)
RCT	Axial	0.742	10.1	0.88	12.2
	coronal	0.683	8.3	0.791	7.7
PT	Axial	0.691	5.6	0.775	6.1
	coronal	0.726	4.4	0.789	3.7
RCT	Average	0.784	8	0.853	9
PT		0.767	4.2	0.784	4.1
R2*	cortex	0.739	5.8	0.757	5.7
	medulla	0.776	8.3	0.802	7.5
ADC	cortex	0.708	5.7	0.873	6.5
	medulla	0.747	7.2	0.88	7.8
FA	cortex	0.724	9	0.829	8.9
	medulla	0.79	10.5	0.792	13

ICC: intra-class correlation coefficient; CoV: Coefficient of Variation; RCT: Renal cortical thickness; PT: Parenchymal thickness.

## Discussion

As at the time of this research, there was apparently no reproducibility study of RCT and PT measurements on MRI in healthy subjects, findings from our study showed that these variables are highly reproducible. In addition, an excellent reproducibility of R2*, ADC and FA in both the cortex and medulla of healthy subjects was observed in our study, and they were similar with what has been reported in literature, that is the between session CoVs of R2*^11,16^, ADC^11,17^ and FA^17^.

The kidney size is of great clinical significance, since many diseases are associated with it; and it is influenced by individual variability. In our series, PT, RCT and CPR were decreased after 40 years old, and only the change of PT had statistical significance which is inconsistent with the findings that both RCT and PT decreases with age^18^. The reason for this may be due to the relatively low sample size in our study. Many angiographic studies pointed out that RCT as a morphological marker was sensitive in diagnosing renal vascular diseases^19,20^. Our results may support this because RCT (SD 0.04) is quite invariable in people of different age and sex; and CPR (SD 0.02) seems to be more stable as shown in Table 2 and Fig. 3. This could be useful in detecting early renal morphological abnormalities. We also found that majority of the morphological parameters of males significantly exceeded that of females, which is in concordant with previous CT studies^18,21^; however, like the result of Gourtsoyiannis *et al*.^21^, the significance is lost between sexes when measurements were corrected for body size (BMI). In addition, we found that the upper and lower thickness in coronal plane was significantly larger than the value in axial renal hilum plane, which has not yet been reported in the literature and could be one reason why the mean RCT in our study (0.51 ± 0.04) was smaller than (0.91–0.92) as reported by Mounier-Vehier *et al*.^19^ in contrast-enhanced CT study which measured RCT only at the upper and lower poles.

Tissue hypoxia is considered pivotal in the development and progression of many kidney diseases^22,23^. BOLD-MRI that measures renal tissue deoxyhemoglobin levels voxel by voxel has become a widely used and robust non-invasive method to estimate renal tissue oxygenation^24,25^. This method explores the fact that oxyhemoglobin is diamagnetic, whereas deoxyhemoglobin is paramagnetic which can induce inhomogeneity and increased T2* decay represented by decreased intensity on T2*-weighted images and increased R2* value^26^. Increases in R2* correspond to higher deoxyhemoglobin concentrations and indicate low tissue oxygenation, whereas decreases in R2* indicate high tissue oxygenation. A wide range of renal cortical and medullary R2∗ (T2∗) values have been presented in the literature, where R2* value of the medulla was higher than that of cortex, confirming the significant cortico-medullary gradient of oxygenation^25,27^. The R2∗/T2∗ values presented in our study are in the range of those described in the literature for baseline results^11,16,28^, while other studies have recorded lower^9,29^ or higher^25^ R2∗ values. Our study demonstrated that cortical R2* value was higher in the older group and was positively correlated with age, and there is a trend for increased medullary R2* in old people, similar to previous findings where both cortical and medullary R2* values increased with age^29^ and only medullary R2* values increased with age^30^. However, Gloviczki *et al*.^31^ and Grassedonio *et al*.^13^ did not find correlation with age. The reason for this may be that Gloviczki *et al*.^31^ included middle-aged and elderly patients with pronounced hypertension or atherosclerotic renal artery stenosis in whom the age range is small and renal tissue oxygenation may mainly be influenced by diseases. However, Grassedonio *et al*.^13^ included small number of young and middle-aged healthy subjects with small age range in subjects whose renal tissue oxygenation may not have been altered significantly and each of their ROI covered both the cortex and medulla. Our results indicated that the aging kidney suffered extensive degrees of hypoxia, which is most prominent in the cortex. This has been confirmed by the animal experiment that increased hypoxia has been demonstrated throughout the aged rat kidneys, most prominently in the cortical zone, as detected by hypoxia–sensitive marker pimonidazole^32^. Several potential mechanisms can be postulated, and more than one might be involved: Within the aging kidney, there are important changes to blood vessel structure and function, which may compromise RBF. RBF has been shown to reduce with age, with redistribution of blood from the cortex to the medulla and leading to low oxygen supply to the renal cortex. Decreased glomerular density and glomerulosclerosis with aging seems to preferentially affect the more superficial nephrons^3,4^. Likewise, a pathological study has demonstrated that arteriolar sclerosis seen with aging involves smaller vessels of the cortex primarily, with secondary changes in the arcuate and inter-lobar vessels^33^. In addition, radiological study with aging man has demonstrated that the angiographically demonstrable changes associated with aging progress is in a centripetal pattern, with the more peripheral portions of vascular bed showing the earliest changes^34^. As to the blood vessel function in aging kidney, there are intrinsic causes of decreased RBF that is the aging kidney has diminished responsiveness to vasodilators and increased sensitivity to vasoconstrictors^4^.

The cortical R2* value was found to be significantly higher in males compared with females, which is consistent with the findings of Pruijm *et al*.^35^ however, other studies didn’t see a difference between males and females^12,13,30^. Our result suggests that cortical oxygenation might be regulated differently in men and women, and it may provide some clues why renal function declines faster in men. A possible explanation for this is that atherosclerosis, increasing oxidative stress with ageing and vascular nitrous oxide (NO) deficiency in males are more pronounced than in females, and are possibly androgen-induced^36,37^. Although there was a trend of higher medullary R2* value in male group, no significant difference was observed between different the two sexes. Possible reason for this may be that it is too weak to be detected with the data used in this study or it is merely due to limited number of samples.

DWI is a non-invasive method to detect the displacement of water molecules within the architecture of tissue that can be quantified from ADC. As one of the simplest parameters reflecting tissue microstructure, the ADC is an overall measurement of water diffusion and microcirculation in the tissue. It is now known that DWI is particularly sensitive to alterations in the renal interstitium, for instance, renal fibrosis, cellular infiltration (inflammatory or tumorous) or edema, intrarenal perfusion and in water handling in the tubular compartment^10^. Some studies have showed a decreased ADC in CKD and obstructive renal diseases^38,39^, which may be the results of worsen renal function (i.e. decreased water reabsorption that cause a lower rate of water transfer across interstitial space) and renal fibrosis. The renal ADC value integrates both the effects of capillary perfusion and water diffusion in the extravascular space. Some literature has demonstrated that the measured ADC is affected by the b-values adopted. Using only high b-value (500–1000 s/mm^2^) will result in a low measured ADC which approximates the true diffusion; while applying only low-b values (0–100 s/mm^2^) will result in a high calculated ADC which reflects both perfusion and diffusion. When a wide range of b-values are used, it will provide the least variation^40^. Thus, 3 b values were used in our series to balance the “T2 shine-through” and capillary effects. Our results are comparable to that of Cox^11^ and Thoeny^40^. However, Cutajar^17^ reported higher ADC than ours, which may be due to the fact that they only used two b-values (0 and 400 s/mm^2^). Furthermore, DW images with b = 0 were used in our study to locate renal cortex and medulla, and we found that ADC value of cortex exceeded that of medulla with statistical significance. The reason for this could be faster water molecule transporting in the cortical glomerulus. We found that medullary ADC value was positively correlated with parenchymal thickness, which may be explained by the reason that kidneys with thicker renal parenchyma have lesser nephrosclerosis, tubular atrophy and renal fibrosis, and better renal function. Similar to the study by lavdas *et al*.^14^, our study did not find dependence of ADC values with age and sex, a recent study in 137 healthy participants did find that ADC values in the kidney may be age- and gender-dependent^15^.

DTI is a development from diffusion-weighted MRI, it can provide insight into the structural properties of tissue by assessing the directionality of water diffusion which is quantified as the percentage of spatially oriented diffusion signal [fractional anisotropy (FA), 0% = complete isotropy; 100% = complete anisotropy]. Diffusion anisotropy is related to structural organization and therefore could be compromised in a pathological process. It was first used by Ries *et al*.^41^ in the kidneys of healthy volunteers, and some studies showed that FA values may aid in the detection of renal microstructural abnormalities in patients^42,43^. DTI studies including ours have demonstrated that diffusion anisotropy in the medulla is considerably higher than in the cortex^41,44,45^, and the tractography reveals a radial diffusion orientation. The reason for this was presumably due to a well-defined renal structure with tubules, collecting ducts and vessels radially oriented towards the pelvis and in which molecules move in a preferential direction. In addition, our study demonstrated that medullary FA decreased with age, which implied structural and functional changes of renal medulla in aging kidneys and may be explained by the aging-related process of potential vascular abnormalities, reduced tubular flow rate, tubular ultrastructural damage (e.g. tubular dilation and atrophy), and interstitial fibrosis^3,4^.

There are several limitations in this study. First, the number of subjects enrolled was relatively small, which may limit the statistical analysis. Further studies with larger sample sizes are needed to further validate the results. Second, GFR and other MRI modalities that measure RBF and perfusion were not performed in this study, this may help in the interpretation of our results. We did not investigate the physiological influence of hydration, furosemide or 100% O2 breathing on renal tissue oxygenation represented as R2*. Third, the pathological results of aging kidney such as decrease in RCT, PT and increase in arteriosclerosis, glomerulosclerosis, tubular atrophy, interstitial fibrosis, and so on were not collected for correlation. Further experimental or clinical studies with pathological findings will be useful to reveal the specific mechanism of R2*, ADC and FA changes in people with different age and sex.

In conclusion, this paper has outlined a multiparametric MRI acquisition to assess the renal morphology and function, and it has shown that PT, cortical R2* and medullary FA values are age dependent and RCT and cortical R2* values are sex dependent. Age- and gender-related effects should be considered in future multiparametric MRI studies using normal values from health controls.

## Materials and Methods

### Study population

The study was carried out in accordance with the principles of the Declaration of Helsinki. Thirty-three healthy subjects (16 males and 17 females; mean age, 38.5 ± 15.7 years) with no history of renal disease, hypertension, diabetes, vascular disease, malignant tumors or medication use were recruited from January 2018 to June 2018. The spot urine samples tested negative for proteinuria by dipstick. In addition, patients were excluded if they had any degree of renal artery stenosis or a history of percutaneous or surgical renal intervention (including angioplasty, renal artery stenting, or transplantation). Other exclusion factors included multiple renal cysts, polycystic kidney disease, hydronephrosis, a unilateral kidney, and inadequate MRI quality for the reason of extensive respiratory motion. The participants were divided into groups (<40 years and >40 years, male and female). Written informed consent was obtained from all the participants after having full understanding of our study which was approved by the ethics committee of Sichuan Cancer Hospital and Institute.

### Imaging

All the subjects were scanned during the morning from 10:00–11:00 am using a 3.0-Tesla MR whole-body scanner (Magnetom Trio, Siemens Medical solution, Erlangen, Germany) with a six-channel flexible body matrix coil. Prior to their MR scans, it was ensured that participants had fasted for at least 4 hours and refrain from smoking for at least 12 hours.

At first, in-phase and opposed-phase images in axial and coronal plane were taken covering both kidneys as scout images (TR/TE/FA = 180 ms/2.2–3.57 ms/65° and 95 ms/2.5 ms/70°; slice thickness 5.0 mm; resolution 1.4 × 0.9 × 3.0 mm). Then, BOLD MRI was obtained with an end-expiration breath-holding of 48 s. The multiple gradient-recalled echo sequence [TR/TE/FA/bandwidth = 90 ms/2.97~40.22 ms/40°/360 Hz/Px; matrix 320 × 320; field of view (FOV) 34~38 cm; concatenations 3; slice thickness 5.0 mm] was used to acquire 8 sets of T2*-weighted images in coronal planes, and quantitative T2* maps were calculated on the scanner. DW MRI was acquired with free breathing, which was not respiratory triggered, and the scan time was 97 s. The echo-planar imaging (EPI) technique and fast gradient sequence (TR/TE/bandwidth/=1800 ms/82 ms/1396 Hz/Px; FOV 34–38 cm; phase direction H>>F; slice thickness 4.0 mm; concatenations 2; phase oversampling 50%; matrix 128 × 128; plus 12 diffusion direction and 3 b-values 0, 300, 600 s/mm^2^) were used to acquire diffusion trace weighted images in the coronal planes, and the average ADC, FA color and tensor maps were subsequently created automatically on the scanner. The average ADC map was calculated from all b values with a monoexponentially fitting model which includes contributions from both diffusion and perfusion. Furthermore, to determine the between session variability of MRI measures, a subset of 15 subjects (age 24–64 years, BMI 19.0–29.4 kg/m^2^) repeated the MRI scans 3 days later.

### Image analysis

**Figure 5:**
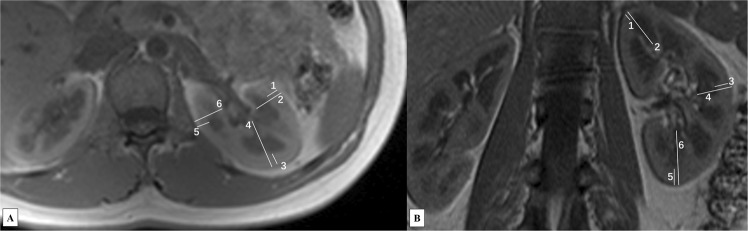
The renal cortical and parenchymal thickness were measured in axial (**A**) and coronal (**B**) plane, respectively. Note: Number 1, 3 and 5 in both axial (**A**) and coronal (**B**) represent cortical thickness, and number 2, 4 and 6 represent parenchymal thickness.

None of the images were excluded because of artifacts and transferred to the workstation (VE31A SL02P10 SMMWP sp02, syngo MultiModality Workplace) for analysis. First and foremost, two professional radiologists (more than 5 years of experience) independently analyzed all the images of the first MRI scans and recorded the measurements. Then, one of the experienced radiologists analyzed all the images of the repeated MRI scans. In each kidney, RCT and PT were measured in T1-weighted images at the anterior, lateral and posterior area in axial planes, and the upper pole, middle and lower pole in coronal planes, respectively (Fig. 5). All the parameters were measured at the level of renal hilum, and CPR was acquired by the ratio of RCT to the nearest parallel PT. For each orientation, the three measurements were averaged together for each kidney and then averaged across kidneys.

Analysis of the maps for BOLD and DW imaging was performed. In each kidney, 6 circular regions of interest (ROIs) with a fixed 21-voxel diameter were manually placed in the upper, middle and lower poles of bilateral renal cortex and medulla for the measurement of R2*, ADC, and FA values (Fig. 1). The visible blood vessels, renal pelvis, cysts, tissue boundaries and areas of heterogeneous signal intensity were avoided. T2* value was obtained in T2* maps, then an average R2* value (=1/T2*) was determined for each ROI. ROIs were drawn on the diffusion weighted images (b = 0 s/mm^2^) to exhibit adequate anatomic details. Then, they were automatically matched on ADC and FA maps for quantification. Diffusion tensor maps were imported to Neuro 3D workstation for analysis.

All the average MRI parameters for each kidney were calculated from averaging the measurements of each kidney by the two radiologists, and the average values of each subject were calculated from the measurements of both kidneys.

### Statistical analysis

All statistical analyses were performed with SPSS software (SPSS 17.0, Chicago, IL, USA). The results were expressed as the mean ± standard deviation (SD). Paired t-test was used to compare the MRI parameters between the left and right kidney. One-way analysis of variance (ANOVA) followed by Student-Newman-Keuls (SNK) test was performed to compare different areas of RCT and PT in the kidney. The baseline characteristics and MRI parameters were compared between different sex and age groups using independent sample t-test; and the mean R2*, FA and ADC values were also compared between renal cortex and medulla with the same method. In addition, Pearson’s correlation analysis was used to detect the correlation between these parameters and associated factors such as height, weight and BMI. To determine the between session and inter-observer variability of MRI measures, the intra-class correlation coefficients (ICCs, average measures, two-way random, absolute agreement) and coefficient of variation (CoV; defined as the standard deviation/mean) were assessed. A two-tailed p value of <0.05 was considered statistically significant in all analyses.
